# Triplex-forming oligonucleotides as an anti-gene technique for cancer therapy

**DOI:** 10.3389/fphar.2022.1007723

**Published:** 2022-12-21

**Authors:** Chun Li, Zunzhen Zhou, Chao Ren, Yi Deng, Feng Peng, Qiongfen Wang, Hong Zhang, Yuan Jiang

**Affiliations:** ^1^ Department of Rehabilitation Medicine, Mianyang Central Hospital, School of Medicine, University of Electronic Science and Technology of China, Mianyang, China; ^2^ Clinical Medical College and The First Affiliated Hospital of Chengdu Medical College, Chengdu, China; ^3^ Department of Rehabilitation Medicine, Shanghai Fourth People’s Hospital Affiliated to Tongji University School of Medicine, Shanghai, China

**Keywords:** triplex-forming oligonucleotides (TFOs), triple helix formation, oncogenes, anti-gene technique, cancer therapy

## Abstract

Triplex-forming oligonucleotides (TFOs) can bind to the major groove of double-stranded DNA with high specificity and affinity and inhibit gene expression. Triplex-forming oligonucleotides have gained prominence because of their potential applications in antigene therapy. In particular, the target specificity of triplex-forming oligonucleotides combined with their ability to suppress oncogene expression has driven their development as anti-cancer agents. So far, triplex-forming oligonucleotides have not been used for clinical treatment and seem to be gradually snubbed in recent years. But triplex-forming oligonucleotides still represent an approach to down-regulate the expression of the target gene and a carrier of active substances. Therefore, in the present review, we will introduce the characteristics of triplex-forming oligonucleotides and their anti-cancer research progress. Then, we will discuss the challenges in their application.

## Introduction

Numerous gene diseases are related to genome mutations, so the therapeutic strategies that enable targeted editing in genetic disease treatment have attracted more attention (Piotrowski-Daspit et al., 2018). Triplex-forming oligonucleotides (TFOs) can be composed of DNA, RNA, or synthetic base analogs linked by phosphodiester backbones (Chin and Glazer, 2009). TFOs can bind to the major groove of homopurine-homopyrimidine stretches of double-stranded DNA in a sequence-specific manner through Hoogsteen hydrogen bonding to form DNA triplexes (Moser and Dervan, 1987; Häner and Dervan, 1990; Reza and Glazer, 2014). Based on this property, TFOs can be used as tools to study gene regulation, DNA reparation, recombination, and mutagenesis (Giovannangeli and Hélène, 1997). In addition, TFOs can target specific genes to form the DNA triplex structures, so they have been investigated in the therapeutic strategies for gene diseases. Studies have predicted that almost 98% of annotated human genes have at least one TFOs target sequence (TTS) within the promoter/transcribed region, and 87% of TTSs are unique to one gene (Reza and Glazer, 2014; Lauria et al., 2020). A total of 519,971 TTSs can serve as potential target sites (Kaushik Tiwari et al., 2022). The high frequency of TTSs in the human genome provides targets for the design of TFOs as a therapeutic targeting approach in gene diseases. At least in principle, TFOs can serve as gene-targeting agents for the transient control of gene activity and alter gene expression through directed mutagenesis (Moasser, 2007). In fact, besides promoter/transcribed region, triplex sites can be located downstream of the transcription start site, including exons and introns, indicating an increased number of potential target genes for triplex-based strategies (Barre et al., 2000).

RNA interference (RNAi) is an effective mRNA-level intervention technology, but it does not change the genetic code, and gene silencing is transient. In comparison, the advantage of TFOs lies in their ability to target and permanently modify disease-specific genes (Zuin Fantoni et al., 2020). Paquet and colleagues suggested that the quantity of TFOs demanded to obtain a significant inhibition was very lower than that in siRNA, and the inhibition effects of the target gene induced by TFOs can last longer (Paquet et al., 2011). Strobel and colleagues chose the entire candidate region for the Huntington’s disease gene at the tip of human chromosome 4 as a target for testing the recognition and enzymatic cleavage effect of a 16-base TFO, then results showed that sequence-specific TFO recognition of the motif within the human genome, and may facilitate the regulation of human genetics (Strobel et al., 1991). So far, the study and application of TFOs are related to the treatment of Duchenne muscular dystrophy, beta-thalassemia mutation, arthritis, HIV infection, and cancer (Reza and Glazer, 2014). In particular, the target specificity of TFOs combined with their ability to suppress oncogene expression has driven their development as anti-cancer agents (Ricciardi et al., 2014; Pyne et al., 2021). Therefore, in the present review, we will introduce the characteristics of TFOs and their anti-cancer research progress. Then, we will discuss the challenges of TFOs in their application.

## Characteristics of triplex-forming oligonucleotides

**FIGURE 1:**
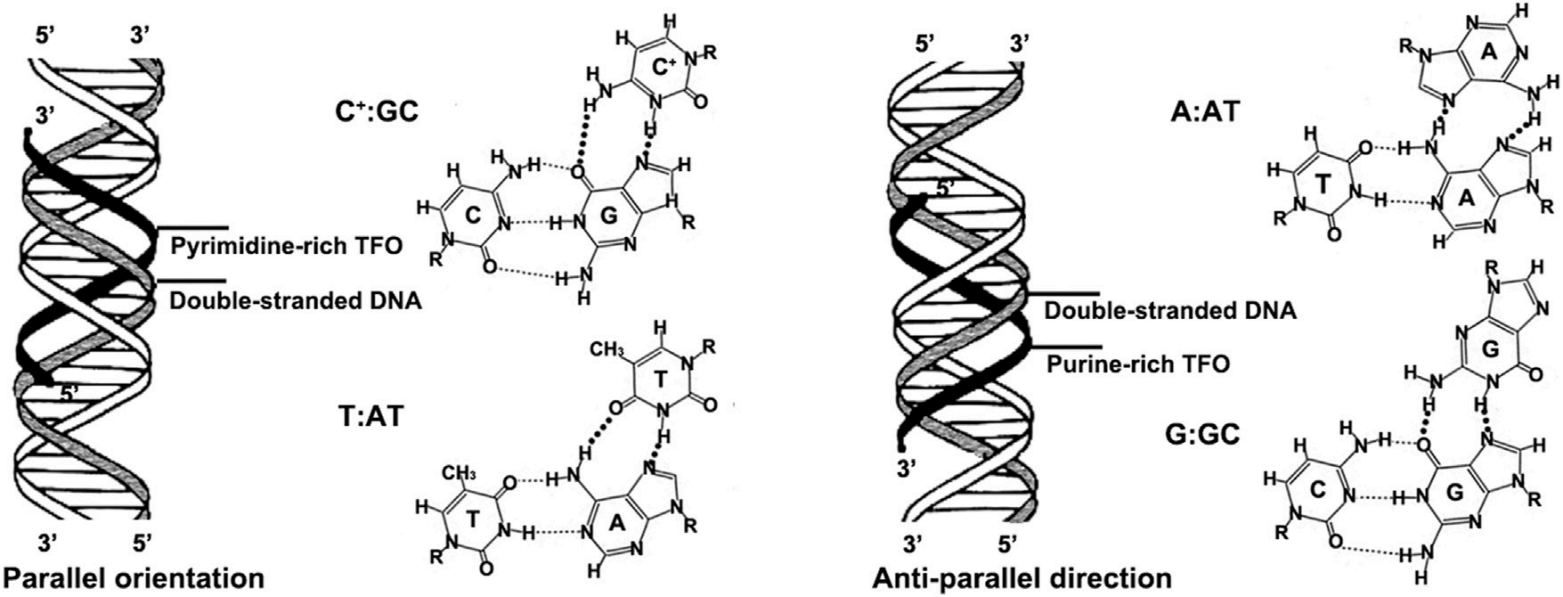
Triplex binding code in the pyrimidine-rich and purine-rich TFO motifs.

TFOs typically are 12–28 long oligonucleotides that bind to specific regions in duplex DNA as a third strand to form a triple helix formation (Beal and Dervan, 1991). The hydrogen bonds involved in triple-helix formation are referred to as Hoogsteen hydrogen bonds and are different from the hydrogen-bonding pattern of the Watson-Crick base pairs (Häner and Dervan, 1990). The Hoogsteen hydrogen bonds can afford the specificity and affinity of the TFOs for their target DNA duplex (Duca et al., 2008). These Hoogsteen bonds are generally weaker than Watson-Crick base pairing but can be stabilized in the presence of divalent cations (e.g., Mg^2+^, Ca^2+^, and Zn^2+^) to enhance triplex stability (Chin et al., 2007; Chin and Glazer, 2009). Moreover, the third-strand binding is kinetically slow compared to Watson-Crick base pairing, but triplexes are stable once formed, exhibiting half-lives on the order of days (Chin et al., 2007; Jain et al., 2008). The formation of triplexes depends on several factors, such as length, divalent cations, temperature, base composition, and the sequence context of the TFO-binding site. In particular, TFO sequence specificity relies on binding to oligopurine-oligopyrimidine target sequences. Two TFO motifs are possible: pyrimidine-rich TFOs bind in a parallel manner, while purine-rich TFOs bind in an anti-parallel orientation. The pyrimidine-rich TFOs consisting primarily of thymines (T) or protonated cytosines (C^+^) bind to DNA with AT and GC base pairs to form the canonical base triads T:AT and C^+^:GC with duplex DNA (Beal and Dervan, 1991; Hennessy et al., 2022). Typically, pyrimidine-rich TFOs can only form the triple-helix formation under acidic conditions. It's due to the N3-protonated cytosines and thymines of TFOs can recognize guanine and adenine to form Hoogsteen-type hydrogen bonds, respectively (Lee et al., 1979; Seio et al., 2020). The purine-rich TFOs consisting primarily of guanines (G) or adenines (A) bind to DNA with AT and GC base pairs in an anti-parallel direction *via* reverse Hoogsteen bonds to form the base triads G:GC and A:AT with duplex DNA (Praseuth et al., 1999; Mukherjee and Vasquez, 2011). Compared to the pyrimidine-rich TFOs, the purine-rich TFOs bind to DNA at neutral pH and require no protonation. However, the guanines in purine-rich TFOs contribute to forming G-quartets in physiologic concentrations of K^+^, and G-quartets presumably reduce the extent of bioavailable TFO. The schematic representation from Figure 1 illustrates the triplex binding code in the purine-rich and pyrimidine-rich TFO motifs. Generally, the oligopurine-oligopyrimidine target sequences are usually located in gene promoter regions, so the TFOs directed against these regulatory sites can selectively reduce transcription of the targeted genes.

## Application research of triplex-forming oligonucleotides for anti-cancer therapy

TFOs were designed primarily for binding the oncogenes and suppressing transcription to reduce cancer cell growth in anti-cancer research. For example, c-myc and bcl-2 are important therapeutic targets. In addition, some of the genes involved in epithelial-mesenchymal transition (EMT) or multidrug resistance (MDR) also can be the target gene.

### c-myc

The c-myc is a small family of proto-oncogenes and is located on human chromosome 8q24.12-q24.13 (Mai and Mushinski, 2003; Mastronikolis et al., 2019). c-myc acts as an essential transcription factor that regulates the expression of many genes involved in cell growth, proliferation, and apoptosis pathways (Liao and Dickson, 2000). c-myc promotes tumor transformation by the induction of genomic instability in critical genes, and amplification and (or) overexpression of the c-myc gene are associated with poor prognosis or decreased survival of patients with cancer (Garte, 1993; Wade and Wahl, 2006). Therefore, seeking therapies targeting the c-myc gene will be key to reversing cancerous growth in patients with cancer. Transcription of the human c-myc initiates from two major start sites designated P1 and P2 (Kim et al., 1998). P2 promoter is the major promoter for c-myc transcription and generates more than 75% of c-myc mRNA (Kim and Miller, 1995). Meanwhile, multiple protein-binding sites are within the P2 promoter. P2 promoter contains a 23 base pair purine-pyrimidine-rich motif (−62 to −40) and is a potential TTS for purine-purine-pyrimidine triplex formation. Kim and colleagues designed a purine-rich TFO targeted to the region of the c-myc P2 promoter and determined the TFO was oriented antiparallel to TTS in a sequence-specific manner and inhibits nuclear protein binding and *in vitro* transcription of the c-myc gene (Kim and Miller, 1995). A previous study first demonstrated that a TFO targeted to a nuclease-sensitive region upstream of the P1 promoter can inhibit c-myc gene transcription in a cell-free system (Cooney et al., 1988). Therefore, Kim and colleagues suggested that a TFO targeted to either P1 or P2 promoter is unlikely to inhibit c-myc expression completely. In the subsequent study, they used both P1- and P2-targeted TFOs to inhibit c-myc expression in the HeLa human cervical carcinoma cell line at the same time. To investigate the effects of phosphorothioate TFOs on c-myc transcription *in vivo*, they employed a luciferase reporter plasmid containing the cDNA encoding a firefly luciferase gene under the control of an 862-bp human c-myc promoter. After HeLa cells were cotransfected with both P1- and P2-targeted TFOs, the luciferase activity was inhibited by 90%. They suggested that TFOs may represent a gene-specific means of inhibiting c-myc gene expression (Kim et al., 1998). Some scholars found that P2-targeted TFOs can enhance the anti-cancer efficacy of gemcitabine (GEM) *via* TFO-targeted DNA damage-induced unscheduled DNA repair synthesis (UDS) in breast cancer cells (Christensen et al., 2006). They also exhibited superior antitumor activity in a mouse model of human colon cancer after being combined with GEM, suggesting that combining gene-specific TFOs with DNA-targeting drugs represents a more effective treatment strategy for solid tumors in humans (Boulware et al., 2014). Interestingly, certain anti-cancer drugs can be used as triplex stabilizing to improve the stability and biological activity of the P2-targeted TFOs, such as daunomycin (DNM). The attachment of DNM can increase the triplex stability and biological activity of the TFOs without compromising their specificity to c-myc gene. The DNM-conjugated TFOs can inhibit the c-myc gene transcription *in vitro* and reduce c-myc promoter activity in prostate and breast cancer cells while less effective against normal cells (Carbone et al., 2004; Napoli et al., 2006). In recent years, the booming field of nanotechnology provides a more effective strategy for TFOs delivery. Huo and colleagues first used ultrasmall 2 nm tiopronin-covered gold nanoparticles (Au-TIOP NPs) as carriers for P2-targeted TFOs delivery. 5′ amino-modified P2-targeted TFOs were linked to 2 nm Au-TIOP NPs through the carboxyl group of tiopronin. Based on the Au-TIOP NPs smaller than 10 nm that could enter the nucleus, the nanoparticle-conjugated TFOs were more effective at reducing c-myc RNA and c-myc protein in breast cancer cells than free TFOs (Huo et al., 2014). In the subsequent study, Huo and colleagues designed another single-stranded sequence to complementarily hybridize with the tail part of the nanoparticle-conjugated TFOs to block the ability of the TFOs to bind with the c-myc gene and then self-assemble into large-sized sunflower-like structures. The sunflower-like structures possess strong near-infrared (NIR) absorption and photothermal conversion ability. After breast cancer cells have taken up the sunflower-like nanostructures, they stand by in the cell cytoplasm until NIR irradiation. Upon NIR irradiation, the sunflower-like nanostructures could disassemble at the melting point (Tm) and release Au-TIOP NPs to enter the nucleus to help TFOs to achieve the c-myc oncogene silencing. This study provides a new approach for controlling c-myc gene silencing in time and space (Huo et al., 2019).

### Bcl-2

The B cell lymphoma-2 (bcl-2) is one of the most important endogenous anti-apoptotic factors and is located on human chromosome 18q21 (Tomita, 2011). Bcl-2 overexpression will increase the mutation opportunities of other genes, resulting in the deterioration of normal cells into tumor cells. Meanwhile, the Bcl-2 gene increases the number of tumor cells and leads to the further development and proliferation of tumors by inhibiting apoptosis (Goldar et al., 2015). Shen and colleagues found two potential TTSs can bind the corresponding TFOs with high specificity and affinity downstream in the bcl-2 promoter region: from −666 to −647 and from −1113 to −1094 relative to the translation start site. Then they designed the corresponding purine-rich TFOs to inhibit the bcl-2 gene transcription in HeLa human cervical carcinoma cell line. In addition, they attempted to label the conversion-electron-emitter (^99^mtechnetium) to the 3′ end of TFOs *via* histidine to induce DNA double-strand breaks (DSB) when brought close to the DNA strand by TFOs. The formed ^99^mtechnetium-coupled TFOs also bind to the TTS to form stable triplexes and inhibit bcl-2 gene transcription *in vitro*, indicating that TFOs are a promising carrier for gene-radiotherapy and may provide an additional therapeutic method for cancers with bcl-2 overexpression (Shen et al., 2003). In addition to the major transcriptional promoter of bcl-2 gene, Shen and colleagues found that the 3′-untranslated region (3′UTR) of the human bcl-2 gene contains a potential TTS located +1946 to +1963 from the translation start site. 3′UTR also plays an important role in the regulation of gene expression. They demonstrated that the TFOs targeted to the 3′UTR of the bcl-2 gene can inhibit the gene expression in HeLa cells. The mechanism is to form a triple helix in 3′UTR to inhibit transcription elongation and affect the progression of RNA polymerase in its synthesis of the mRNA resulting in mRNA lacking a polyA tail is very unstable and rapidly degraded in Hela cells. They also found that TFO with an amino linkage at the 3′-end alone was particularly resistant to nuclease degradation, which may contribute to maintaining the effect of TFO on bcl-2 expression last for 72 h after transfection (Shen et al., 2001). Taniguchi and Sasaki labeled the non-natural nucleoside analogue, the W-shaped nucleoside analogue (WNA-βT), at the 3′-end of the bcl-2-targeted TFOs to increase the triplex stability. The TFOs with WNA- βT exhibited a higher antiproliferative effect than the corresponding natural TFOs in human A549 lung cancer cells. They suggested that the TFOs incorporating artificial nucleoside analogues may be powerful antigene agents for inhibition of the bcl-2 gene expression (Taniguchi and Sasaki, 2012).

### HER2

The human EGF-like receptor 2 (HER2) is functionally implicated in the pathogenesis of human breast cancer and is an important prognostic and predictive marker in breast cancer (Moasser, 2007; Tapia et al., 2007). HER2 is located on human chromosome 17q12-21.3, encoding a transmembrane growth factor receptor with tyrosine kinase activity. The receptor protein interacts with HER family members and their ligands to regulate cell growth, differentiation, and proliferation through signal transduction (van de Vijver, 2001). HER2 overexpression promotes tumorigenesis that making HER2 is suitability as a drug target in cancer drug development. Kaushik and colleagues reported a TFO-based therapeutic strategy for targeting HER2 gene amplifications (Kaushik Tiwari et al., 2022). They designed two purine-rich TFOs (HER2-1 and HER2-205) to target the polypurine sequence in the promoter region of the HER2 gene at positions −218 to −245 relative to the transcription start site and the coding region beginning at position 205, respectively. When TFOs bind to the target polypurine sequence within the major groove of the double helix in an anti-parallel direction to form the DNA triplex structures will induce DNA perturbation that can impede replication fork progression, resulting in fork collapse and double-strand break (DSB) formation. The formation of DNA triplex structures makes sufficient DNA damage to activate apoptosis in breast cancer. Compared with the control group, TFOs (HER2-205) treatment of HER2-positive breast cancer xenografts resulted in a 52% reduction in tumor volume, equivalent to the 58% reduction observed with trastuzumab. It indicates that activating the DNA damage response with TFOs treatment is as effective as the administration of trastuzumab. They also tried to use biodegradable nanoparticles (NPs) to serve as a delivery platform. For example, PLA-HPG was screened from a copolymer of poly (lactic acid) (PLA) and hyperbranched polyglycerol (HPG). The TFOs encapsulated PLA-HPG NP delivery system can significantly improve the efficacy of TFO treatment. These findings offer a feasible therapeutic strategy for targeting tumors with HER-positive.

### Ets-2

E26 oncogene homolog 2 (Ets-2) is a member of the ETS transcription factor family and is located on human chromosome 21q22. The abnormal expression of Ets2 exists in various cancer types, including prostate, breast, and thyroid cancers. It may contribute to the occurrence and progression of tumors by activating the expression of multiple genes that promote cell proliferation and invasion or prevent apoptotic cell death. Carbone and colleagues found a potential TTS located −40 to −65 relative to the transcription start site in the Ets2 promoter, which contains a region that can be bound to transcription factors of the Sp family. Sp1 sites may be critical for promoter activity of the Ets2 gene. They designed a pyrimidine-rich Ets2-targeting TFO, which has high affinity and specificity for the TTS, for inhibiting the bind of Sp1/Sp3 transcription factors and Sp1 site *in vitro*. They found that Ets2-targeting TFO can act as a selective transcriptional repressor of Ets2 transcription in human prostate cancer cells, suggesting that the triplex formation was the functional mechanism of the anti-transcriptional activity of the Ets2-TFO (Carbone et al., 2003). The pre-initiation complex (PIC) is formed by the assembles of RNA polymerase II (Pol II) and general transcription factors, like the TFIID complex, which contains the TATA-binding protein (TBP) and TBP-associated factors (TAFs), and is essential for the initiation of transcription. Carbone and colleagues suggested that Sp1 can interact with the TAFs proteins, such as TAFII130 and TAFII250, and then promote their assembly into PIC as a tethering moiety. Therefore, they further demonstrated that Ets2-targeting TFOs could prevent the binding of Sp1, TAFII130, and TAFII250 to the Ets2 promoter and interfere with the assembly of PIC. Those results further explain the potential mechanism of triplex DNA-mediated transcriptional repression in cancers with Ets-2 overexpression (Jain et al., 2009).

### Other

Some of the genes involved in EMT or MDR have also drawn the concerns of many researchers. EMT is a process that epithelial cells lose the apical-basal polarity and cell-cell adhesion and then transit to invasive mesenchymal cells. It is well-known that EMT plays an important role in cancer progression, metastasis, and even drug resistance (Du and Shim, 2016). The c-met gene encodes a receptor tyrosine kinase receptor (RTK), also commonly referred to as mesenchymal-epithelial transition factor (MET), and its ligand is the hepatocyte growth factor (HGF) (Faiella et al., 2022). The binding of MET to HGF contributes to the regulation of EMT. Singhal and colleagues designed a purine-rich TFO to bind the TTS located −144 to −119 relative to the transcription start site in the c-met promoter. The TTS contains the binding site of the transcription factor Sp1, and any mutation in this Sp1 binding site will inhibit the c-met transcription. They found that the c-met targeted TFOs lead to cell death and tumor regression in hepatoma, representing a promising tool for inhibiting cancer cell proliferation (Singhal et al., 2011). P-glycoprotein (P-gp/MDR1) is a multidrug efflux transporter that protects cells by exporting xenobiotics out from the plasma membrane to the extracellular space. The overexpression of P-gp as a cause of multidrug resistance is a key player in the multidrug-resistant phenotype in cancer (Callaghan et al., 2014; Pokharel et al., 2017). Based on the MDR1 gene (ABCB1) encoding P-gp, Stierlé and colleagues synthesized a DNM-conjugated TFO to pass the cell membrane, remain in DNM-resistant cell lines, and then inhibit the expression of the MDR1 gene and P-gp (Stierlé et al., 2008).

## Challenges of triplex-forming oligonucleotides

The experiments described in the previous section have shown that TFOs can be used as antigene agents to control the activity of the tumor-associated gene. But many challenges remain, particularly efficient uptake of TFOs into the nucleus, stability once inside the cell, and specific, high-affinity binding to TTS in a cellular environment.

### Design of TFOs

Pyrimidine-rich TFOs require a protonated cytosine to form triplexes under acidic conditions, while guanine-rich TFOs tend to form G-quartets under physiological K^+^ concentrations, which has restricted their use as gene-targeting agents. Moreover, a quick degradation of TFOs also is a big drawback. Therefore, modifications are necessary for the TFOs design. Rusling introduced the nucleobase 6-amino-5-nitropyridin-2-one (Z) to take the place of cytosine in pyrimidine-rich TFOs to overcome the pH dependence of triplex formation (Rusling, 2021). In guanine-rich TFOs, the presence of N, N-diethylethylene-diamine-modified bases may also reduce G-quartet formation (Reza and Glazer, 2014). Torigoe and colleagues reported 2′-O,4′-C-aminomethylene-bridged nucleic acid (2′,4′-BNA(NC)) modification of TFOs significantly increased the nuclease resistance of TFOs and existing good stability in human serum (Torigoe et al., 2011; Torigoe et al., 2012). The TFO backbone can also be modified to improve intracellular resistance to nucleases, such as N,N-diethylethylenediamine (DEED), phosphoramidate, phosphorothioate, methylphosphonate or a peptide-like backbone to form so-called peptide-nucleic acids (PNAs) (Chin and Glazer, 2009; Reza and Glazer, 2014; Chandrasekaran and Rusling, 2018). In addition, the low stability of the triplex and limitations of the target DNA sequence are the major drawback in triplex formation by a natural TFO (Obika, 2004). TFOs inhibit transcription initiation through competition with transcription binding to DNA, but unmodified oligonucleotides may not form stable enough triplex complexes with their DNA target for the arrest of transcription elongation. Mayer and colleagues suggested that introducing the pyrrolidino isocytidine pseudonucleoside and the corresponding phosphoramidite building block into TFOs enhanced the DNA affinity due to the positive charge at the pyrrolidino subunit (Mayer et al., 2005). Faria and colleagues reportped that covalent attachment of an intercalating acridine derivative at the 5′ end of the TFOs inhibited transcriptional elongation in cells, indicating that improvement of triplex stability by modifications increases the intracellular efficacy of triplex-induced effects (Faria et al., 2000). The above modifications are smart and appropriate, but the influence on the biophysical characteristics and activity of TFO *in vitro* and *in vivo* needs further research.

### Efficient delivery of TFOs

TFOs must be effectively delivered to and taken up by cancer cells to significantly regulate the target oncogene phenotype and reduce off-target and toxicity effects. Given the characteristics of cell membrane potential, the addition of positively charged lysine residues can enhance the cellular uptake of TFOs (Ricciardi et al., 2014). Previous studies have reported some strategies for the delivery of TFOs into cells, including nucleofection, cell-penetrating peptides (CPPs), and nanoparticles (Ricciardi et al., 2014). The advantage of nucleofection is the commercially available kits are readily available, but can only be used *in vitro* and may lead to high levels of cell death compared to nanoparticle delivery or other methods. Conjugation of TFOs to CPPs or delivery in NPs contributes to effective transfection with lower cell death and direct *in vivo* delivery, representing a feasible delivery strategy (Ricciardi et al., 2014). But, until now, the Efficient delivery of TFOs into cells remains a challenge.

### Activity of TFOs

Once delivered into the cytoplasm, TFOs can be affected by cellular conditions, such as potassium and magnesium concentrations. Within the nucleus, the charge repulsion between the three polyanionic strands and accessibility of binding sites in a chromosomal context may affect the stable triplex formation (Reza and Glazer, 2014). Moreover, the stable triplex formation also requires contiguous homopurine bases to avoid pyrimidine interruptions within the purine stretch can inhibit triplex formation. Of course, modification can enhance the stability of TFOs but may affect the biological activity of TFOs. For instance, extensive sugar modification can decrease the biological activity of TFOs *in vivo* (Mukherjee and Vasquez., 2011).

## Summary and outlook

Since short oligonucleotides were found that can bind in the major groove of the DNA duplex to form a triple helix formation and play a role in the control of gene expression, many scholars were devoted to exploring the mechanism of TFOs forming triple helix formation and the specific applications of TFOs as an anti-gene strategy during the past decades, especially the identification and manipulation of oncogenes that play a key role in the progression and maintenance of cancers. The application potential of TFOs in therapeutical and biotechnological aspects has been widely investigated. But up to now, they have not been utilized therapeutically. On the one hand, the inherent restrictions of TFOs still exist. Modifications are necessary but can’t cover everything. For instance, modifications can enhance the stability of TFOs but may affect biophysical characteristics and activity. Modification methods may also be complex, and economic costs need to be taken into account. On the other hand, several other gene-targeting methodologies designed to edit DNA are flourishing and aroused more research interest from scholars in recent years, such as zinc-finger nucleases (ZFNs), transcription activator-like effector nucleases (TALENs), and CRISPR/Cas9 platform (Chandrasekaran and Rusling, 2018). In short, TFO-related research seems to be gradually snubbed.

Important to note is that some bioactive substances, such as Auger-electron-emitting radionuclide ^125^I, pyrene, and psoralen, can be conjugated or labeled with TFOs to target specific sites in the genome based on the sequence-specific action of TFOs (Panyutin and Neumann, 1994; Sedelnikova et al., 2000; Li et al., 2007; Benfield et al., 2008; Majumdar et al., 2008; Dahmen et al., 2016). It indicates that TFOs can act as a carrier of active substances. In particular, the conjugation of TFOs to psoralen provides a powerful approach to modifying DNA *in vitro* and in cells (Mukherjee and Vasquez, 2011). Psoralen is a planar triheterocyclic compound containing furan and pyrone rings and can intercalate in DNA, and following exposure to ultraviolet (UVA) irradiation can form interstrand cross-links (ICLs) in double-stranded DNA (Vasquez, 2010). The psoralen-labeled TFOs (pso-TFOs) can be used to induce site-specific DNA damage by directly inactivating the interest gene under the control of UVA irradiation at 365 nm. Thus, pso-TFOs have the potential to irreversibly modify target genes (Barre et al., 2000; Diviacco et al., 2001). The use of pso-TFOs may be limited by UVA does not penetrate internal organs and psoralen potentiates carcinogenesis (Sargent et al., 2011), but it indicates that the covalently linking photoactivatable derivatives to TFOs contribute to achieving the timing of gene damage can be controlled by light. The photoactivation of photoactivatable derivatives-labeled TFOs could have several advantages. First, genetic manipulation could be controlled at the target location and specific time; Second, light irradiation is a clean and safe intervention; Third, the photoinduced targeted gene damage would be highly specific with insignificant toxic side effects (Kolevzon and Yavin, 2010). Photosensitizers (PS) with strong photosensitivity and low toxin may be the potential candidates to replace psoralen for conjugate with TFOs. PS labeled on TFOs may improve the stability of TFOs and achieve the photoinduced targeted gene damage by releasing reactive oxygen species (ROS). Interestingly, some PS also are sonosensitizer (e.g., hematoporphyrin, photofrin, and chlorin e6), which can be activated to generate ROS under ultrasound (Kuroki et al., 2007; Hong et al., 2020; Mai et al., 2020; Zhang et al., 2020). PS-labeled TFOs can be activated by visible light or ultrasound, that may provide advanced technology for clinical applications.

Moreover, the continuous research and innovation of drug delivery systems will provide an appropriate vehicle for the delivery of TFOs, such as nanoparticles, exosomes, and metal-organic frameworks (MOFs), which could be used as an ideal delivery system and would allow systemic injections of TFOs. Therefore, TFOs as an anti-gene technique for cancer therapy still have great attraction and application prospects and need further research and exploration.
